# Red blood cell distribution width in pediatric congenital heart disease: A review of clinical applications

**DOI:** 10.1097/MD.0000000000048937

**Published:** 2026-05-22

**Authors:** Shuqiong Xu, Junru Wang, Jun Yin, Yongyu Cao, Tongyong Luo, Xianmin Wang, Qingsong Wang

**Affiliations:** a Department of Pediatrics, West China Hospital Sichuan University Jintang/Jintang First People’s Hospital, Chengdu, Sichuan, China; b Department of Ultrasound, West China Hospital Sichuan University Jintang/Jintang First People’s Hospital, Chengdu, Sichuan, China; c Pediatric Cardiology Center, Medical Sichuan Provincial Women’s and Children’s Hospital/The Affiliated Women’s and Children’s Hospital of Chengdu College, Chengdu, Sichuan, China.

**Keywords:** cardiac function, congenital heart disease, red blood cell distribution width

## Abstract

Congenital heart disease (CHD) encompasses a heterogeneous spectrum of anatomical lesions ranging from simple shunts to complex cyanotic malformations, representing the most common congenital anomaly in children with variable clinical outcomes. Red blood cell distribution width (RDW), an indicator of erythrocyte volume heterogeneity, has demonstrated associations with cardiac dysfunction across diverse CHD phenotypes. This review synthesizes evidence (2015–2023) from PubMed, Web of Science, Cochrane Library, and China National Knowledge Infrastructure regarding RDW’s clinical utility in pediatric CHD. Current evidence indicates that elevated RDW correlates with disease severity, particularly in heart failure and pulmonary hypertension cohorts, and shows potential for perioperative risk stratification (e.g., prolonged mechanical ventilation). However, reported cutoff values vary substantially across studies (13.9%–16.0%), likely reflecting the anatomical and physiological heterogeneity of CHD populations – wherein RDW elevation in cyanotic lesions may reflect chronic hypoxia-driven erythropoiesis, while in acyanotic shunts it indicates volume overload-mediated inflammation. Existing data predominantly derive from retrospective, single-center studies lacking anatomical stratification, limiting causal inference. RDW serves as an inexpensive, accessible biomarker for risk assessment in resource-limited settings, though interpretation requires contextualization by specific CHD subtype and confounding factors (anemia, nutritional status). Future prospective multicenter studies must establish anatomy-specific reference ranges and validate temporal predictive utility through stratified analyses of simple versus complex lesions, cyanotic versus acyanotic physiology, and preoperative versus postoperative stages.

## 1. Introduction

Congenital heart disease (CHD) is one of the most common congenital malformations in children, with a prevalence of approximately 0.8% to 1.2%.^[1–3]^ Its pathophysiological mechanisms are complex, and clinical manifestations vary, often accompanied by hemodynamic abnormalities, cardiac dysfunction, and pulmonary hypertension (PH), significantly affecting the quality of life and prognosis of affected children. Traditional assessments of cardiac function primarily rely on medical history, symptoms, and physical examination, which are subjective, lack specificity, and may lead to missed diagnoses. Moreover, biomarkers such as B-type natriuretic peptide (BNP) and N-terminal pro-B-type natriuretic peptide (NT-ProBNP) are expensive, limiting their widespread use in routine monitoring and long-term management. Therefore, there is an urgent need for a simple and accessible clinical indicator that can effectively evaluate cardiac function, is associated with surgical outcomes, and guides individualized treatment in children with CHD.

Red cell distribution width (RDW) is an index used to assess the heterogeneity in red blood cell (RBC) volume, originally applied primarily for differentiating types of anemia. However, an increasing body of research has demonstrated the significant clinical value of RDW in the diagnosis and prognostic evaluation of various cardiovascular diseases.^[4,5]^ Elevated RDW is associated with several pathological processes, including inflammation, oxidative stress, and cardiac dysfunction.^[6]^ As these mechanisms are widely present in children with CHD, the application of RDW in CHD has garnered growing attention.

Recent studies^[7–10]^ have revealed significantly elevated RDW levels in children with CHD, closely associated with cardiac function, PH, and prognosis. The mechanisms underlying RDW elevation in CHD may involve multiple factors. First, increased RDW may result from impaired erythropoiesis, bone marrow stress responses, and shortened RBC lifespan, reflecting chronic inflammation, malnutrition, and oxidative stress in children with CHD.^[11]^ In addition, complications such as heart failure (HF) and PH can impair RBC deformability, disrupt microcirculation, and exacerbate tissue hypoxia, further contributing to RDW changes.^[6]^ Notably, elevated RDW is not only a consequence of these pathological states but may also play a role in the onset and progression of cardiovascular diseases. Thus, a thorough investigation of RDW changes in CHD and their relationship with clinical status is crucial for understanding the pathophysiological processes of CHD and developing individualized therapeutic strategies.

The primary objective of this study is to analyze the pathological changes of RDW in children with CHD and to review its clinical utility in assessing cardiac function, diagnosing PH, and predicting postoperative outcomes. By summarizing these insights, this study aims to provide clinicians with an effective and accessible tool to better understand the cardiac functional status and prognostic risks of pediatric CHD patients, thereby optimizing therapeutic strategies and offering a more reliable basis for personalized management in this population.

## 2. Methods

### 2.1. Search strategy and selection criteria

This structured narrative review (distinct from formal systematic review or meta-analysis) systematically searched PubMed, Web of Science, Cochrane Library, and China National Knowledge Infrastructure for relevant articles published between January 2015 and December 2023. The search strategy employed keyword combinations including: “red cell distribution width,” “red blood cell distribution width,” “RDW,” “congenital heart disease,” “congenital heart defect,” “children,” “pediatric,” “heart failure,” “pulmonary hypertension,” “pulmonary arterial hypertension,” “postoperative outcomes,” “mechanical ventilation,” and “prognosis.” Boolean operators “AND” and “OR” were applied to retrieve literature covering associations between RDW and clinical outcomes in pediatric CHD.

Studies were included if they: enrolled children or adolescents (≤18 years) with confirmed CHD; reported RDW values obtained from routine hematological examinations; investigated relationships between RDW and HF, PH, perioperative indicators, or clinical prognosis; and were original clinical studies published in English or Chinese. Exclusion criteria comprised: adult-only populations (≥18 years), non-CHD focus, lack of original clinical data, case reports, conference abstracts with insufficient data, letters, editorials, or duplicate publications.

Two reviewers independently screened titles and abstracts, followed by a full-text review of potentially relevant articles. Disagreements were resolved through discussion and consensus.

### 2.2. Data extraction

A standardized form was used to extract the following information: study design (retrospective/prospective), country of origin, sample size, patient demographics (age range, sex distribution), CHD phenotypes (e.g., simple shunts vs complex cyanotic lesions), timing of RDW measurement (preoperative vs postoperative), RDW-related definitions or cutoff values, major clinical outcomes (mortality, mechanical ventilation duration, intensive care unit [ICU] stay), and principal study findings.

### 2.3. Synthesis of evidence

Because the included studies differed substantially in CHD anatomical subtypes (simple vs complex lesions), age distributions, timing of RDW measurement relative to surgery, laboratory analyzer platforms, and outcome definitions, quantitative pooling (meta-analysis) was not performed. Instead, evidence was synthesized narratively with particular attention to: methodological limitations including confounding factors (anemia, nutritional deficiencies, inflammatory status, transfusion history); heterogeneity of RDW cutoff values across studies; and distinction between associative relationships and true predictive performance. This approach aligns with guidelines for narrative reviews of heterogeneous clinical evidence.

## 3. The pathological changes of RDW in children with CHD

### 3.1. Impaired erythropoiesis and microcirculatory disorders

One important mechanism for elevated RDW is impaired erythropoiesis, which may be caused by bone marrow stress responses, anemia, and abnormal regulation of erythropoietin (EPO).^[12]^ In children with CHD, complications such as HF and PH lead to chronic hypoxia, which stimulates the bone marrow to increase erythropoiesis. The RBCs produced under these stress conditions are often immature and vary greatly in size, leading to increased RDW.^[13]^ Furthermore, HF and inadequate circulatory oxygen supply reduce EPO synthesis, impair erythropoiesis, and subsequently increase erythrocyte heterogeneity, which results in higher RDW values.

Children with CHD typically experience nutritional and metabolic disorders, such as deficiencies in iron, folate, and vitamin B12. Nutritional deficiencies directly affect RBC production and maturation, leading to variations in RBC size. In the case of HF, reduced blood flow to the gastrointestinal tract impairs nutrient absorption, particularly of essential nutrients such as iron and vitamin B12, which are crucial for erythropoiesis. Malnutrition and anemia further disrupt the production and development of RBCs, contributing to the elevation of RDW.^[14]^

The deformability of RBCs is a key characteristic that allows them to pass through microcapillaries in the circulatory system. Elevated RDW often indicates changes in the volume and morphology of RBCs, leading to decreased deformability. When RDW is elevated, the differences in RBC volume increase, and the cells become stiffer, making them more likely to be damaged and rupture in the microcirculation. This microcirculatory disturbance exacerbates tissue hypoxia, increases systemic inflammation, and creates a vicious cycle that further elevates RDW.^[15]^ In children with CHD, changes in hemodynamics and inadequate tissue oxygenation further reduce RBC deformability, resulting in a significant increase in RDW.

Crucially, the mechanisms described in this subsection – including the links between hypoxia, EPO dysregulation, and erythropoietic stress – are predominantly extrapolated from studies in adult cardiovascular disease or noncardiac pediatric conditions, rather than being directly proven in children with CHD. Furthermore, although RDW is often associated with altered RBC deformability, RDW is fundamentally a measure of anisocytosis (heterogeneity in cell volume), not a direct marker of cellular rheology. The conceptual distinction between RDW and RBC deformability should be clearly recognized to avoid overinterpretation of RDW as a specific indicator of microcirculatory dysfunction.

### 3.2. Inflammatory responses and immune regulation

Chronic inflammation is a critical mechanism underlying changes in RDW in children with CHD. Complications such as HF and PH induce systemic inflammation, leading to elevated levels of inflammatory cytokines, such as tumor necrosis factor and interleukin-6. These cytokines suppress EPO synthesis, inhibit bone marrow hematopoiesis, and result in abnormal RBC production.

In addition, inflammation disrupts the stability of RBC membranes, accelerates RBC destruction, and increases heterogeneity in cell volume, which contributes to elevated RDW. Under chronic inflammatory conditions, oxidative stress may also damage RBCs by promoting lipid peroxidation in cell membranes, altering membrane flexibility, and increasing the likelihood of rupture. Together, inflammation and oxidative stress impact RBC lifespan, leading to greater variability in cell size and elevated RDW.^[16]^

### 3.3. Neuroendocrine activation and circulatory abnormalities

In children with CHD, chronic cardiac dysfunction and hypoxic conditions can lead to abnormal activation of the neuroendocrine system, particularly the sympathetic nervous system and the renin-angiotensin-aldosterone system. This activation causes vasoconstriction, elevated blood pressure, and reduced renal perfusion, further impairing EPO production and affecting bone marrow erythropoiesis, which contributes to increased RDW.^[17]^ In addition, heightened sympathetic activity may destabilize RBC membranes, accelerating their rupture and release, thereby increasing heterogeneity in RBC volume.

In summary, elevated RDW is not only a result of these pathological states but may also adversely impact cardiac function in CHD patients, leading to further disease progression. Increased RDW reflects abnormalities in RBC volume and function, exacerbating microcirculatory resistance and reducing tissue oxygenation. These changes in oxygenation can negatively influence cardiac function, promoting myocardial hypoxia, metabolic disturbances, and worsening HF. The interplay between RDW and cardiovascular disease creates a vicious cycle, where continuous RDW elevation indicates ongoing cardiac deterioration and poor prognosis.

### 3.4. Methodological limitations and confounding factors

Despite the aforementioned associations, the application of RDW as a cardiac-specific biomarker in children with CHD is constrained by significant methodological limitations. The RDW value is highly susceptible to multiple noncardiac confounding factors; thus, its elevation in this population often reflects a composite of pathological and technical influences rather than a direct manifestation of cardiac dysfunction.

First, nutritional and hematological abnormalities are prevalent in children with CHD and serve as major confounders. Iron deficiency anemia, frequently resulting from inadequate dietary intake, malabsorption due to intestinal congestion secondary to HF, or iatrogenic blood loss from frequent phlebotomy, is a common cause of elevated RDW. Furthermore, deficiencies in vitamin B12 or folate – often observed in postoperative or feeding-intolerant children – exacerbate ineffective erythropoiesis, thereby increasing RBC volume heterogeneity.^[18]^ Second, inflammatory states significantly interfere with RDW interpretation. Active infection or postoperative systemic inflammatory response syndrome can elevate RDW by suppressing bone marrow erythropoiesis and shortening RBC survival. Consequently, multivariate analyses typically require adjustment for inflammatory markers such as high-sensitivity C-reactive protein (hs-CRP) to validate the independent predictive value of RDW.^[19]^ Third, technical variability at the detection level cannot be overlooked. Differences in automated hematology analyzers, reagent batches, or sampling timing can cause fluctuations in RDW results, compromising consistency in clinical interpretation.^[20]^

In conclusion, unless these confounding factors (e.g., anemia type, nutritional status, inflammatory indices) are rigorously identified and adjusted for in statistical models, the observed correlation between RDW and cardiac dysfunction “may merely reflect underlying nutritional deficiencies, inflammation, or technical artifacts, rather than true myocardial pathology.” This characteristic significantly undermines the reliability of RDW as a standalone, cardiac-specific biomarker and emphasizes the necessity of comprehensive clinical evaluation combined with multiparameter correction.

## 4. The application of RDW in children with CHD4

### 4.1. RDW and cardiac function in children with CHD

In pediatric CHD research, RDW has attracted significant attention, and its association with cardiac function has been validated in numerous studies. Liu et al^[15]^ found that RDW was correlated with cardiac function in children with CHD, particularly closely related to HF in this population. Kaya et al^[21]^ also confirmed that RDW levels in children with CHD increased with the severity of heart dysfunction. Nagaoka et al^[22]^ investigated the impact of RDW in school-age children with CHD. In their study, which involved RDW testing and analysis of 293 school-aged children with CHD, they found a positive correlation between RDW and the severity of heart disease – specifically, the higher the RDW, the greater the severity of the cardiac condition. Furthermore, Li et al^[23]^ demonstrated that RDW levels were significantly elevated in patients with HF compared to healthy controls. This increase in RDW levels was positively correlated with the degree of cardiac dysfunction, suggesting that RDW may serve not only as an adjunctive indicator for assessing heart function but also as a supportive tool in the assessment of HF. Zhu et al,^[24]^ after conducting an in-depth study on the relationship between RDW and HF and its severity, concluded that RDW levels were significantly elevated in HF patients compared to the control group. Further analysis of RDW’s value in assessing the risk of HF revealed an area under the receiver operating characteristic (ROC) curve of 0.857, indicating excellent diagnostic performance. Specifically, the sensitivity of this method was 83.00%, and the specificity was 79.30%, which robustly supports the significant and practical value of RDW in helping identifying and diagnose HF. Another study^[25]^ also indicated that RDW not only reflects the severity of HF but also plays a crucial role in its diagnosis, risk stratification, and monitoring treatment, highlighting its potential utility in evaluating HF. In addition, Alshawabkeh et al^[20]^ noted that elevated RDW could serve as an independent risk factor for all-cause mortality in CHD. These findings collectively indicate a strong association between RDW and cardiac function in children with CHD, underscoring its clinical application value in heart function assessment.

HF is a common complication in children with CHD, with a complex pathophysiological mechanism involving structural and functional abnormalities of the heart. Changes in RDW may reflect increased cardiac workload and an imbalance between oxygen supply and demand. Fukuta et al^[17]^ first established a close relationship between RDW and BNP in cardiovascular diseases, suggesting that RDW can be used to assess cardiac dysfunction.

In recent years, the strong association between RDW and acute heart failure (AHF) in children has been thoroughly validated. RDW levels increase as AHF severity worsens, and can indirectly reflect changes in NT-proBNP levels and left ventricular ejection fraction. RDW is therefore a convenient and useful marker for assessing the severity of AHF in children and for monitoring cardiac function.^[7]^ Another study^[26]^ showed a positive correlation between NT-proBNP levels and the severity of HF in children with CHD, with higher NT-proBNP levels correlating with more severe HF. NT-proBNP can assist in assessing intracardiac shunting and volume overload in CHD patients. A study by Yang et al^[27]^ found that RDW and NT-proBNP levels were significantly higher in children with CHD and HF compared to those without HF. In ROC curve analysis, RDW levels >13.90% were identified as a risk threshold that may help identify HF in children with CHD. This suggests that RDW elevation aligns with changes in NT-proBNP, and both markers are valuable for HF diagnosis.

RDW is also closely related to chronic heart failure (CHF). A study by Cauthen et al^[28]^ found that baseline RDW levels and continuous increases in RDW were associated with poor long-term prognosis in CHF patients. Similarly, Felker et al^[12]^ demonstrated that RDW-induced hematologic abnormalities were strongly correlated with the incidence of complications and mortality in CHF patients, and RDW could serve as an independent prognostic marker in this population. Elevated RDW and NT-proBNP levels are useful in diagnosing CHF in children with CHD, with NT-proBNP being valuable for evaluating disease severity. However, whether RDW can serve as a marker for the severity of CHF in CHD patients remains uncertain.^[26]^ Another study^[29]^ showed that in children with HF, a critical RDW threshold of 16.4% was significantly correlated with fractional shortening. RDW can serve as a simple and easily measurable marker for assessing HF in children prior to echocardiographic examination.

These clinical observations are physiologically plausible, as the elevated RDW levels in HF patients likely reflect the underlying pathological milieu of chronic inflammation, impaired erythropoiesis, and persistent tissue hypoxia commonly seen in CHD. In conclusion, RDW plays a significant role in evaluating cardiac function in children with CHD and can also serve as a biomarker for screening and diagnosing HF.

### 4.2. RDW and pulmonary arterial hypertension in children with CHD

The correlation between RDW and pulmonary arterial hypertension (PAH) in children with CHD has increasingly gained attention. Studies by Zhu et al^[30]^ and Zhang et al^[31]^ have shown that RDW is an important prognostic factor in idiopathic PAH and an independent risk factor for predicting patient mortality. Zhu^[32]^ further emphasized that children with CHD and pulmonary hypertension (PAH-CHD) have significantly higher RDW levels than those with CHD alone, while their left ventricular ejection fraction is notably lower. Notably, when RDW exceeds 14.8%, the risk of death in CHD-PH patients increases significantly, with a sensitivity of 66.7% and specificity of 77.0% for predicting mortality.

Research by Gursoy et al^[33]^ also demonstrated that PH in PAH-CHD patients is significantly correlated with CRP, the neutrophil-to-lymphocyte ratio, RDW, and other markers. A study by Jin et al^[34]^ clearly showed that in children with PAH-CHD, especially those with HF, RDW and NT-proBNP levels are significantly elevated. Furthermore, another study indicated that RDW values in children with PAH-CHD who died were significantly higher than in those who survived, and RDW levels provide an important reference value for determining whether CHD children have HF.^[26]^ In addition, compared to the non-PH group, the RDW and NT-proBNP levels in the PH group were significantly elevated. ROC curve analysis showed that RDW >14.55% is a risk threshold for identifying PAH in CHD patients, and RDW levels are directly correlated with pulmonary arterial pressure in these children.

In conclusion, RDW appears to be a valuable clinical indicator for evaluating and monitoring children with CHD and PAH. It correlates with disease severity and is associated with poor prognosis, serving as an independent risk factor for mortality. However, the reported RDW cutoff values varied significantly across studies, likely due to differences in study population, age distribution, CHD subtype, clinical endpoints, laboratory platforms, and adjustment for confounders. Therefore, these thresholds should not be directly generalized across different clinical settings. These findings emphasize the importance of monitoring RDW dynamics in the diagnosis and treatment of children with CHD to facilitate the early identification and intervention of PAH.

## 5. Perioperative applications of RDW in pediatric CHD

### 5.1. RDW and prolonged mechanical ventilation

In the perioperative period of heart interventions or surgeries in children with CHD, RDW plays a significant role in disease assessment, complications, and prognostic evaluation.^[12,28]^ A study by Yin et al^[5]^ found a close correlation between preoperative RDW and the platelet-to-lymphocyte ratio in CHD-PAH patients. These 2 indicators were associated with early postoperative complications and clinical outcomes, and could identify the likelihood of prolonged mechanical ventilation following heart surgery to some extent.

Another study^[35]^ further confirmed that preoperative RDW elevation in CHD patients was associated with prolonged mechanical ventilation time, ICU stay, and overall hospital stay after surgery. This suggests that RDW can assist in early identification of high-risk patients who may face complications during the perioperative period. Research by Otero et al^[36]^ divided patients into 2 groups based on RDW values: normal range (RDW = 11.5%–14.5%) and high level (RDW > 14.5%). Even after adjusting for factors like age, gender, and BMI, patients with elevated RDW were 32% less likely to be off mechanical ventilation within 30 days after surgery compared to those with normal RDW. This correlation was further supported by research from Schepens et al,^[37]^ which found that patients in the fifth quintile of RDW had a 2.59-fold higher risk of requiring mechanical ventilation compared to those in the first quintile. In addition, a study by Massion et al^[38]^ revealed that among children undergoing CHD surgery, preoperative RDW was significantly higher in patients who died during the postoperative hospitalization compared to survivors. Preoperative RDW was also strongly correlated with postoperative ICU stay and total hospitalization time in these patients.

The mechanistic basis for this association may involve hypoxemia-driven erythropoietic stress, endothelial dysfunction, and systemic inflammation, which are cardinal features of PAH pathophysiology. In summary, RDW is closely related to the duration of postoperative mechanical ventilation in CHD patients. By assessing RDW levels preoperatively, clinicians can better identify the need for respiratory support following surgery and take early intervention measures to improve the prognosis of these children.

### 5.2. RDW and postoperative prognosis

Postoperative recovery following congenital heart surgery is a complex process that involves multiple physiological factors. In children with CHD, RDW levels are considered an effective indicator for evaluating postoperative recovery. High preoperative RDW levels are associated with poorer postoperative prognosis in critically ill CHD patients, and persistent elevation of RDW after surgery is typically linked to slower or poor recovery.^[5]^ A study by Osadnik et al^[20]^ reported that in children undergoing CHD interventions, elevated RDW levels were associated with a significantly higher incidence of restenosis after surgery, and preoperative RDW elevation could affect the prognosis of critically ill CHD patients.^[39]^

In children with CHD-PAH, preoperative RDW and platelet-to-lymphocyte ratio are closely correlated with early postoperative complications and clinical outcomes.^[7]^ Furthermore, a study by Cauthen et al involving 6159 ambulatory patients with CHF demonstrated that baseline RDW levels were significantly associated with long-term all-cause mortality.^[20]^ In their multivariate Cox proportional hazards analysis, which adjusted for key covariates including age, hemoglobin, BNP, and renal function, each 1% increment in baseline RDW was associated with a 9% increase in mortality risk (risk ratio = 1.09, 95% confidence interval = 1.01–1.17; *P* = .029). Notably, the study identified an RDW cutoff of ≥16% as a robust predictor of poor prognosis. Patients with an RDW ≥16% at baseline exhibited a markedly higher mortality rate compared to those with RDW <16%, with the risk escalating to more than fivefold in certain analytical subsets (*P* < .0001 for trend). These findings provide a rigorous statistical basis for utilizing RDW ≥16% as a threshold for risk stratification in relevant patient populations. RDW was also significantly positively correlated with ICU stay (*P* < .0001) and total hospitalization time (*P* < .0001). This correlation was more pronounced in non-cyanotic CHD patients, particularly those without anemia, where RDW was more strongly associated with clinical outcomes. Therefore, preoperative RDW may serve as a valuable indicator to identify poor prognosis in children with CHD undergoing surgery, particularly in non-anemic patients, where it may reflect underlying inflammatory stress.

In a clinical data analysis of 2230 CHD surgical patients,^[15]^ RDW levels were classified into 3 categories: RDW < 13.6%, 13.6% ≤ RDW < 14.5%, and RDW ≥ 14.5%. The results showed that as RDW levels increased, the in-hospital mortality risk also increased, with each increase in RDW category associated with a 0.85-fold higher in-hospital mortality rate. This study confirmed that RDW levels are positively correlated with in-hospital mortality and length of hospital stay, and that combining RDW with a severity score provided better predictive value for in-hospital mortality than using the score alone. Previous studies^[40]^ have shown that younger and more complex CHD patients are more likely to have cardiovascular abnormalities, and such patients are more likely to undergo aggressive surgical or interventional treatment. In addition, they found that in infants with CHD, especially those with HF, earlier surgical treatment was associated with higher survival rates.

In summary, elevated preoperative RDW is closely associated with postoperative complications, in-hospital mortality, and hospital stay in children with CHD. Crucially, these perioperative associations should be interpreted within the correct pathophysiological context: preoperative RDW elevation often reflects a preexisting vulnerability to inflammatory or nutritional disturbances rather than acting as a direct causal driver of postoperative events. Therefore, monitoring RDW changes is of significant clinical importance for identifying postoperative recovery, evaluating prognosis, and making timely adjustments to treatment plans.

## 6. Limitations of current evidence

Beyond the aforementioned findings, several methodological constraints warrant careful consideration to contextualize the current evidence base. The existing literature on RDW in pediatric CHD is predominantly composed of retrospective or cross-sectional studies with single-center designs, which inherently limit causal inference and generalizability due to wide variations in sample size and a lack of adequate power for subgroup analyses based on distinct CHD phenotypes (e.g., simple shunts vs complex single-ventricle physiology). Crucially, this aggregation of anatomically and physiologically distinct lesions into a single “CHD” category obscures potentially divergent RDW pathophysiology: RDW elevation in cyanotic conditions (e.g., tetralogy of Fallot) likely reflects chronic hypoxia-driven erythropoietic stress, whereas in acyanotic shunt lesions (e.g., large ventricular septal defect), it may predominantly indicate volume overload and inflammatory activation. Similarly, the prognostic implications of RDW may differ between preoperative, post-corrective, and palliated stages, yet existing data rarely stratify analyses by these clinically relevant subgroups, limiting the actionable specificity of current evidence. Furthermore, substantial heterogeneity exists regarding RDW measurement protocols, including the timing of blood sampling, the type of automated analyzers used, and the absence of standardized quality control, all of which may introduce laboratory variability. This issue is compounded by insufficient control for key confounding factors, as few studies have adequately adjusted for nutritional status, iron studies, or inflammatory markers – variables that are critically intertwined with RDW values in this population. The inclusion of literature from the China National Knowledge Infrastructure database, while broadening the scope, may introduce language bias, and the overall evidence is likely subject to publication bias with an underrepresentation of gray literature and negative results.

Temporally, the absence of prospective longitudinal studies leaves unresolved whether RDW changes truly precede clinical events or merely reflect preexisting disease states. Without prospective evidence confirming that RDW alterations antedate adverse outcomes, its role as a true predictive biomarker remains largely speculative. Finally, it is important to acknowledge that the proposed mechanistic links between RDW and CHD – such as hypoxia, inflammation, and EPO suppression – are largely extrapolated from adult cardiovascular or noncardiac pediatric literature, highlighting a significant gap in direct mechanistic evidence specific to children with CHD. Addressing these limitations through future multicenter, prospective, and mechanistically focused investigations is essential for validating RDW’s clinical utility.

## 7. Summary and outlook

RDW, as an inexpensive and universally available hematological parameter, has emerged as a promising composite indicator of systemic physiological stress in pediatric CHD. Unlike specialized biomarkers such as NT-proBNP or troponin, which primarily reflect myocardial wall stress or injury, RDW captures the integrated effects of chronic hypoxia, inflammatory activation, erythropoietic dysfunction, and nutritional deficiencies – all prevalent in the CHD population. Current evidence suggests that elevated RDW correlates with cardiac decompensation, pulmonary vascular remodeling, and adverse perioperative outcomes across the heterogeneous CHD spectrum, from simple shunt lesions to complex cyanotic malformations. However, the clinical interpretation of RDW requires anatomical and physiological contextualization: values must be evaluated within specific subgroups (cyanotic vs acyanotic, preoperative vs post-corrective) and adjusted for confounding factors, including iron deficiency and inflammatory status. When applied with these considerations, RDW serves not as a standalone diagnostic tool but as a complementary risk-stratification adjunct that enhances the accessibility of prognostic assessment, particularly in resource-limited settings where advanced biomarker testing or sophisticated imaging is unavailable.

However, the long-term clinical effectiveness of RDW as a prognostic marker in CHD requires further validation through large-scale, multicenter studies. Future research should prioritize the development and validation of multivariable risk prediction models that integrate RDW with established biomarkers and advanced imaging parameters. Specifically, these models must rigorously adjust for key confounding factors – such as anemia, nutritional deficiencies, active infection, and transfusion history – to isolate the independent prognostic value of RDW. Crucially, to bridge the gap between association and causality, future investigations should employ prospective, multicenter cohort designs to validate the temporal predictive value of RDW and establish age-specific and CHD-subtype reference ranges that explicitly stratify by cardiac anatomy (simple shunts vs complex single-ventricle physiology), cyanotic status, and surgical stage (preoperative vs postoperative). Such stratification is essential given that the pathophysiological interpretation of RDW elevation differs markedly between cyanotic lesions (primarily reflecting chronic hypoxia-driven erythropoietic stress) and acyanotic shunt defects (primarily indicating volume overload-mediated inflammation and cardiac decompensation), across different laboratory platforms. Furthermore, such studies should perform head-to-head comparisons between RDW and conventional biomarkers (e.g., NT-proBNP, troponin, and CRP), assess the incremental value of combining RDW with ultrasound parameters, and incorporate health economic analyses to evaluate the cost-effectiveness and practical utility of RDW-guided strategies in resource-limited settings, ultimately contributing to improved management strategies in pediatric CHD. Such advancements in research will pave the way for more precise and individualized treatment strategies, ultimately enhancing the care and management of children with CHD.

## Acknowledgments

We acknowledge all the authors in this article.

## Author contributions

**Formal analysis:** Shuqiong Xu, Jun Yin.

**Resources:** Shuqiong Xu, Junru Wang, Xianmin Wang.

**Data curation:** Junru Wang, Qingsong Wang.

**Investigation:** Junru Wang, Jun Yin, Yongyu Cao.

**Writing – original draft:** Junru Wang, Jun Yin, Qingsong Wang.

**Project administration:** Jun Yin, Qingsong Wang.

**Methodology:** Tongyong Luo, Xianmin Wang.

**Supervision:** Tongyong Luo, Xianmin Wang.

**Validation:** Tongyong Luo, Xianmin Wang.

**Writing – review & editing:** Tongyong Luo, Xianmin Wang.

**Conceptualization:** Xianmin Wang, Qingsong Wang.
